# Severity of adipose tissue dysfunction is associated with progression of pre-diabetes to type 2 diabetes: the Tehran Lipid and Glucose Study

**DOI:** 10.1186/s12889-023-17381-1

**Published:** 2024-01-09

**Authors:** Mohammad Jalali, Zahra Bahadoran, Parvin Mirmiran, Fereidoun Azizi, Farhad Hosseinpanah

**Affiliations:** 1 Nutrition and Endocrine Research Center, Research Institute for Endocrine Sciences, Shahid Beheshti University of Medical Sciences, No 23, A’rabi St, Yeman Av, Velenjak, Tehran, Iran; 2 Department of Community Nutrition, School of Nutrition and Food Sciences, Shiraz University of Medical Sciences, Shiraz, Iran; 3 Department of Clinical Nutrition and Dietetics, Faculty of Nutrition Sciences and Food Technology, National Nutrition and Food Technology Research Institute, Shahid Beheshti University of Medical Sciences, Tehran, Iran; 4 Endocrine Research Center, Research Institute for Endocrine Sciences, Shahid Beheshti University of Medical Sciences, Tehran, Iran; 5 Obesity Research Center, Research Institute for Endocrine Sciences, Shahid Beheshti University of Medical Sciences, Tehran, Iran

**Keywords:** Adipose tissue, Visceral adiposity index, Pre-diabetes, Type 2 diabetes

## Abstract

**Background:**

The association of prediabetes (Pre-DM) regression and progression with visceral adiposity index (VAI) and adipose tissue dysfunction (ATD) remains to be investigated.

**Methods:**

The present cohort study was conducted within the framework of the Tehran Lipid and Glucose Study (TLGS) on 1458 Pre-DM cases (aged ≥ 21 years) who were followed for nine years. VAI was estimated based on waist circumference, body mass index, triglycerides, and high-density lipoprotein cholesterol. ATD status (i.e., absent, mild-moderate, and severe) was defined based on the age-stratified cutoff values of VAI. Multinomial logistic regression models with adjustment of potential confounders were used to estimate the chance of Pre-DM regression to normoglycemia or progression to T2D across ATD status.

**Results:**

During the study follow-up, 39.0% of the participants developed T2D, and 37.7% returned to normoglycemia. Compared to mild-moderate ATD, Pre-DM subjects with severe ATD had a higher risk of developing T2D by 45% (OR = 1.45, 95% CI = 11.08–1.93). Severe ATD was also associated with a decreased chance of returning to normoglycemia by 26% (OR = 0.74, 95% CI = 0.55–0.99). Participants with severe ATD had significantly higher fasting (overall mean = 111, 95% CI = 109–112 vs. 106, 95% CI = 105–108 mg/dL) and 2h-serum glucose (overall mean = 165, 95% CI = 161–168 vs. 153, 95% CI = 149–156 mg/dL) concentrations over time.

**Conclusion:**

Severe ATD was associated with an elevated risk of developing T2D and longitudinal poor-glycemic controls in Pre-DM subjects. ATD may be a simple and useful index for detecting subjects at a higher risk of Pre-DM progression to T2D, allowing for timely intervention strategies.

## Introduction

Pre-diabetes (Pre-DM) is an intermediate state of hyperglycemia, diagnosed by either impaired fasting glucose (IFG), impaired glucose tolerance (IGT), or combined IFG-IGT [1]. Pre-DM will progress to type 2 diabetes (T2D) in 25% of participants during three-five years, and 70% of Pre-DM subjects are expected to develop T2D within their lifetime [2, 3]. Although obesity is considered an independent risk factor of pre-DM progression to T2D, it remains inconclusive which obesity measure can predict Pre-DM regression and progression better [2, 4, 5]. Compared to waist circumference, a reduction of more than five% in body weight and body mass index showed more significant associations with regression of both Pre-DM and T2D; in contrast, reduction in neck circumference (i.e., a marker of upper body subcutaneous fat) was not associated with regression of Pre-DM and T2D [4]. Excess visceral fat but not general adiposity was independently associated with incidence of Pre-DM and T2D; during a median seven years of follow-up, each one kg-weight gain was independently associated with risk of T2D by 6%, with no associations for body mass index, total body fat, or subcutaneous fat [5].

Although body mass index (BMI) has traditionally been used as a surrogate measure of adiposity, body fat distribution strongly influences the development of glucose intolerance and T2D [5, 6]. Excessive visceral fat, but not general adiposity, was independently associated with the risk of pre-DM and developing T2D [5]. Visceral adiposity index (VAI), an empirical mathematical sex-specific model composed of anthropometric and lipid measures validated by magnetic resonance imaging (MRI), accurately estimates the visceral adiposity accumulation and dysfunction associated with cardiometabolic risk [7–10]. VAI showed a strong correlation with both area and volume of visceral adipose tissue (VAT), insulin sensitivity, and impaired glucose metabolism [7, 9]. VAI is supposed to provide a simple proxy measure of functional and structural adiposity; several documents introduced VAI as a surrogate of adipose tissue dysfunction (ATD) [11]. The appropriate age-stratified cut-off points of VAI associated with cardiometabolic risk, validated by Amato et al., are used to identify different states of ATD [12].

Although not established, VAI has been supposed to be a strong predictor of T2D among healthy adults [13–15]. Potential predictive values of VAI and ATD status with the progression or regression of Pre-DM remained to be elucidated by population-based studies. To address this knowledge gap, we aimed to assess the possible association of ATD status (estimated by measuring VAI) with the chance of regression to normoglycemia or progression to T2D within nine years, in a cohort of middle-aged Pre-DM men and women, using data from a well-known population-based cohort, the Tehran Lipid and Glucose Study (TLGS). A practical implication of the study finding would be using ATD as a simple and useful index for detecting subjects at a higher risk of Pre-DM progression to T2D, allowing for timely intervention strategies.

## Subjects and methods

### Study design and population

#### TLGS study design and population

Details of study design and study population (sample size estimation, sampling methods, study subjects recruitment, baseline assessments and follow-up examinations) have been described previously [16]. The TLGS, an ongoing prospective population-based study performed on a representative sample of the Tehran (the capital of Iran) population, aims to determine the prevalence of and prevents the non-communicable disease (NCD) risk factors [17]. The baseline survey was performed in 1999–2001 (phase one) among more than 15,000 residents (aged ≥ 3 years) of district-13 of Tehran. All measurements were done at baseline and all subsequent examinations with three-y follow-up intervals. The same standard approach was followed for data collection across consecutive examinations of the TLGS.

### Current study design and population

**Fig. 1:**
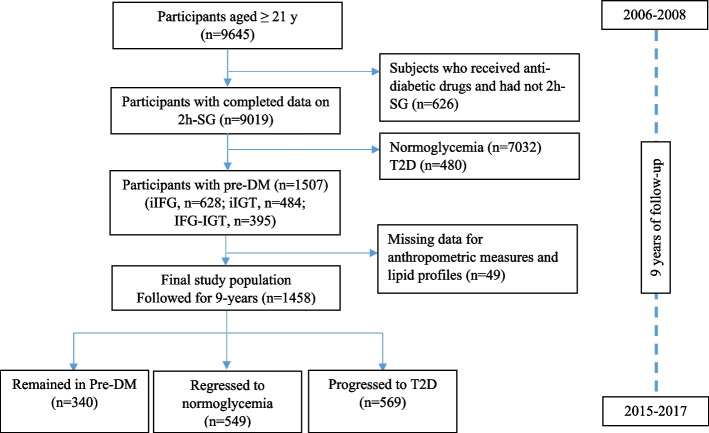
The study flowchart

The current study used the data from 9645 individuals aging 21 years or older at the third examination of the TLGS (2006–2008). After exclusion of healthy participants (*n* = 7032), participants with prevalent T2D (*n *= 480), and those with missing data regarding two-hour post challenge serum glucose (2h-PCSG) (*n* = 626), anthropometric and lipids (*n* = 49), 1458 participants diagnosed with Pre-DM remained eligible to be followed within three consecutive phases (phase four: 2009–2011, phase five: 2012–2014, and phase six: 2015–2017) for a median of nine years (see Fig. 1). All of the study participants assigned the informed written consent form. The protocol of current study was conducted based on the Declaration of Helsinki and approved by the ethics committee of the Research Institute for Endocrine Sciences, Shahid Beheshti University of Medical Sciences (IR.SBMU.ENDOCRINE.REC.1402.019).

### Assessments of exposure, outcomes, and covariates

Trained interviewers collected the populations’ information using a pretested questionnaire including sociodemographic (e.g. sex, date of birth, smoking status, anthropometric measures, physical activity) and medical data (family history of disease, and medications). Details of anthropometric measurements (i.e., body weight, height, BMI, and waist circumference) [18, 19], measurement of systolic blood pressure (SBP) and diastolic blood pressure (DBP) [20], physical activity, [21] at baseline (2006–2008) have been reported by the TLGS research group elsewhere. Details of biochemical measurements, including serum glucose concentration, standard 2h-PCSG test, high-density lipoprotein cholesterol (HDL-C), and serum TG have been described in detail elsewhere [22]. The intra and inter-assay coefficients of variation were both < 5% for the measurements.

We defined VAI as:

$$\mathrm{in}\;\mathrm{Male}=\left(\frac{\mathrm{WC}\left(\mathrm{cm}\right)}{39.68+\left(1.88\times\mathrm{BMI}\right)}\right)\times\left(\frac{\mathrm{TG}\left(\frac{\mathrm{mmol}}{\mathrm L}\right)}{1.03}\right)\times\left(\frac{1.31}{\mathrm{HDL}-\mathrm c\left(\frac{\mathrm{mmol}}{\mathrm L}\right)}\right)$$

$$\mathrm{in}\;\mathrm{Female}=\left(\frac{\mathrm{WC}(\mathrm{cm})}{36.58+\left(1.89\times\mathrm{BMI}\right)}\right)\times\left(\frac{\mathrm{TG}(\frac{\mathrm{mmol}}{\mathrm L})}{0.81}\right)\times\left(\frac{1.52}{\mathrm{HDL}-\mathrm c\left(\frac{\mathrm{mmol}}{\mathrm L}\right)}\right)$$

Assuming VAI = 1 in healthy normal-weight subjects with normal adipose distribution and normal TG and HDL levels [7]. ATD status (i.e., absent, mild-moderate, and severe ATD) was defined based on the age-stratified cutoff values of VAI for cardiometabolic diseases [12].

All Pre-DM subjects were assessed for the occurrence of the outcomes at three-year intervals (2009–2011, 2012–2014, or 2015–2017) during the study follow-up. Having at least one of the IFG (100 ≤ FSG < 126 mg/dL) or IGT (140 ≤ 2 h-SG < 200 mg/dL) was considered as Pre-DM [23]. The first occurrence of both normal fasting glucose (NFG, i.e., FSG < 100 mg/dL) and normal glucose tolerance (NGT, i.e., 2 h-SG < 140 mg/dL) was defined as returning to normal glycemia. Meeting at least one of the following state was defined as T2D: FSG ≥ 126 mg/dL or 2 h-SG ≥ 200 mg/dL, or self-reported use of glucose-lowering medications [23]. Family history of T2D (FHD) was defined as a self-reported positive having at least one parent or sibling with T2D.

### Statistical methods

Statistical analyses were conducted using the SPSS for Windows version 20 (SPSS Inc., Chicago, IL, USA). Findings on covariate variables are expressed as means (SD), median (inter-quartile range, IQR) or percentages for continuously distributed and categorical variables, respectively.

Potential confounding variables were selected from literature [24] and confirmed by the statistical evidence [25]. A univariate analysis was performed for potential confounding variables, and those with P_E_ < 0.2 were selected for the final multivariable model; P_E_ (*P*-value for entry) determines which variables should be included in the multivariable model [25]. Finally, three models were conducted including age- and sex-adjusted model, and two multivariable adjusted models: second model (additionally adjusted for and FSG and FHD) and third model, additionally adjusted for smoking and physical activity. Because our outcomes are common in the study population (> 10%), the adjusted odds ratios (ORs) derived from the logistic regression can no longer approximate the relative risks (RRs) [26]. The ORs (95% CIs) of the final models were, therefore, converted by the following formula to approximate the RR to adjust for outcomes incidence: RR = OR ÷ [(1-P_0_) + (P_0_ × OR)], in which P_0_ indicates the incidence of the outcome of interest in nonexposed group (i.e., the incidence of the outcome in the mild-intermediate ATD group in this study) [26].

To determine longitudinal changes of glycemic parameters (FSG and 2 h-SG concentrations) over time, and to estimate their cumulative average across ATD status (mid-moderate *vs.* severe ATD), repeated-measures ANOVA was used.

## Results

During a median nine-years follow up of 1458 (mean age of total population: 53.0 ± 13.7, 46.8% men) pre-DM participants of the TLGS, we documented 569 cases with T2D incidence (39.0%) and 549 cases of normoglycemia (37.7%), and 340 (23.3%) subjects were remained Pre-DM. About 55% (*n* = 802) of the participants were categorized as mild-moderate ATD and 45% (*n* = 656) as severe ATD at baseline. Baseline demographic and clinical characteristics of the study patients across the outcomes are shown in Table 1. Participants who regressed to normoglycemia during the study follow-up were more likely to be younger, had lower values of BMI, WC, SBP and TG-to-HDL-c ratio, and lower FSG, and 2 h-SG levels. Participants with new onset T2D tended to have higher baseline of VAI compared with those who remained Pre-DM or regressed to normoglycemia (4.0 ± 2.8 vs. 3.6 ± 2.5 and 3.3 ± 2.5, respectively).

**Table 1:** Baseline characteristics of the study participants across the final glycemic status (*n* = 1458)

	Remained Pre-DM (*n* = 340)	Regressed to normoglycemia (*n* = 549)	Progressed to T2D (*n* = 569)
Age (y)	55.6 ± 13.4	50.2 ± 14.7^a,b^	54.3 ± 12.5
Men (%)	51.2	42.2	45.5
FHD (%)	17.4	17.6	24.2
Medications			
Lipid-lowering (%)	6.2	4.9	7.4
BP-lowering (%)	8.8	6.7	9.8
Current smoker (%)	12.3	11.8	9.1
PA (MET-min/week)^c^	1582 (704–2801)	1854 (1100–3223)^b^	1764 (931–3222)
BMI (kg/m^2^)	29.0 ± 4.9^a^	28.4 ± 4.6^a^	30.3 ± 5.3
WC	98.0 ± 11.7	94.9 ± 11.3^a,b^	99.7 ± 10.7
SBP (mmHg)	124 ± 19	120 ± 19^a,b^	126 ± 19
TG-to-HDL ratio	5.1 ± 3.4	4.6 ± 3.6^a^	5.5 ± 4.1
FSG (mg/dL)	101 ± 7.8^a^	97.5 ± 8.8^a,b^	104 ± 9.3
2 h-SG (mg/dL)	135 ± 30.3^a^	130 ± 30.8^a^	148 ± 30.1
VAI	3.6 ± 2.4	3.3 ± 2.5^a^	4.0 ± 2.8
Mid-moderate ATD (%)	56.8	61.9^a^	47.8
Severe ATD (%)	43.2	38.1^a^	52.2

Analysis of variance was used with Bonferroni post hoc test

*T2D* Type 2 diabetes, *FHD* Family history of T2D, *BMI* Body mass index, *WC* Waist circumference, *FSG* Fasting serum glucose, *2 h-SG* 2-h serum glucose, *TG* Serum triglyceride, *HDL-C* High-density lipoprotein cholesterol, *VAI* Visceral adiposity index, *BP* Blood pressure, *SBP* Systolic blood pressure, *MET* Metabolic equivalent, *PA* Physical activity

Data are mean ± SD or percent

^a^Significant difference with T2D and

^b^Significant difference with Pre-DM (*P* < 0.05)

^c^Median (inter-quartile rage, IQR)

The odds ratio (95% CI) of Pre-DM regression to normal glycemia and progression to T2D in relation to the status of ATD are presented in Table 2. Compared to mild-moderate ATD, Pre-DM subjects who had severe ATD had a higher the risk of developing T2D by 45% (OR = 1.45, 95% CI = 11.08–1.93) independent of the well-established risk factors of T2D; the estimated RR from adjusted-OR was 1.15 (95% CI = 1.03–1.31). Conversely, severe ATD significantly decreased chance of returning to normoglycemia by 26% (adjusted-OR = 0.74, 95% CI = 0.55–0.99; the estimated RR from adjusted OR = 0.89, 95% CI = 0.81–0.99).

**Table 2:** The odds ratio (95% CI) of prediabetes (Pre-DM) regression to normoglycemia and progression to type 2 diabetes (T2D) in relation to adipose tissue dysfunction (ATD)

	**Regressed to normoglycemia**		**Progressed to T2D**	
ATD	Mild-moderate	Severe	Mild-moderate	Severe
Crude	1.00	0.81 (0.61–1.06)	1.00	1.43 (1.09–1.88)
Model 1	1.00	0.74 (0.55–0.98)	1.00	1.38 (1.04–1.81)
Model 2	1.00	0.75 (0.56–1.00)	1.00	1.41 (1.06–1.87)
Model 3^a^	1.00	0.74 (0.55–0.99)	1.00	1.45 (1.08–1.93)

Data are ORs and 95% CI

Multinomial logistic regression was used

Model 1, adjusted for age and sex

Model 2, additionally adjusted for fasting serum glucose (FSG) and family history of T2D

Model 3, additionally adjusted for smoking and occupation

^a^The expected RRs from the obtained adjusted-ORs [26] was 0.89 (95% CI = 0.81–0.99) for normoglycemia for severe ATD. For T2D, the expected RRs from the obtained adjusted-ORs was 1.15 (95% CI = 1.03–1.31) for severe ATD

Table 3 displays the mean (SD) of FSG and 2h-SG concentrations across ATD status during the study follow-up. Trends of FSG and 2h-SG changes over time were significantly different between two levels of ATD (P_time×group_ = 0.001 and 0.01, respectively). Participants with severe ATD had a significant higher FSG (overall mean = 111, 95% CI = 109–112 vs. 106, 95% CI = 105–108 mg/dL, *P* = 0.043) and 2h-SG (overall mean = 165, 95% CI = 161–168 vs. 153, 95% CI = 149–156 mg/dL, *P* = 0.020) concentrations over time.

**Table 3:** Mean FSG and 2h-SG concentrations (mg/dL) over 9 years of follow-up across baseline-ATD status

**ATD status**	**Baseline** (2006–2008)	**First follow-up** (2009–2011)	**Second follow-up** (2012–2014)	**Third follow-up** (2015–2017)	**Overall mean** (95% CI)	**P **_**time×group**_
**FSG**						
Mild-moderate ATD	101 ± 9.3	106 ± 19.4	109 ± 22.8	110 ± 28.2	106 (105–108)	0.001
Severe ATD	102 ± 9.3	111 ± 23.1	115 ± 29.3	116 ± 33.0	111 (109–112)	
**2h-SG**						
Mild-moderate ATD	135 ± 32.4	142 ± 50.1	158 ± 59.9	176 ± 69.6	153 (149–156)	0.010
Severe ATD	142 ± 30.4	153 ± 55.0	171 ± 68	192 ± 76.0	165 (161–168)	

Data is reported based on mean ± SD

The repeated measurement ANOVA was used

*FSG* Fasting serum glucose, *2 h-SG* 2-h serum glucose

## Discussion

This study is the first demonstration of the performance of ATD for predicting the risk of developing T2D or the chance of returning to normoglycemia among Pre-diabetics. During a nine-year follow-up within a well-characterized cohort study, we observed that severe ATD (i.e., defined based on age-stratified cutoff values of VAI for cardiometabolic diseases [12]) in the pre-DM subjects significantly reduced their chance of returning to normoglycemia and increased risk of progression to T2D. The Pre-DM subjects with severe ATD, compared to mid-moderate ATD, also experienced worse longitudinal fasting glucose and postprandial glucose tolerance (i.e., measured as repeated FSG and 2h-SG levels during the study with 3-year intervals), glycemic parameters mainly denote pancreatic β-cell function and peripheral insulin sensitivity [27]. The severity of ATD appears to be used by clinicians as a simple and practical measure to pick up and target high-risk subjects to delay and prevent Pre-DM progression. Independent of body weight reduction, redistribution of adipose tissue shifting from visceral to subcutaneous fat and decreased ectopic fat deposition may be a potential therapeutic target for Pre-DM subjects to reduce the risk of T2D [28–30]. Pharmaceutical approaches, including treatment with peroxisome proliferator-activated receptor γ (PPARγ) agonists like troglitazone, efficiently improve ATD by decreasing visceral and increasing subcutaneous fat [29, 30].

**Fig. 2:**
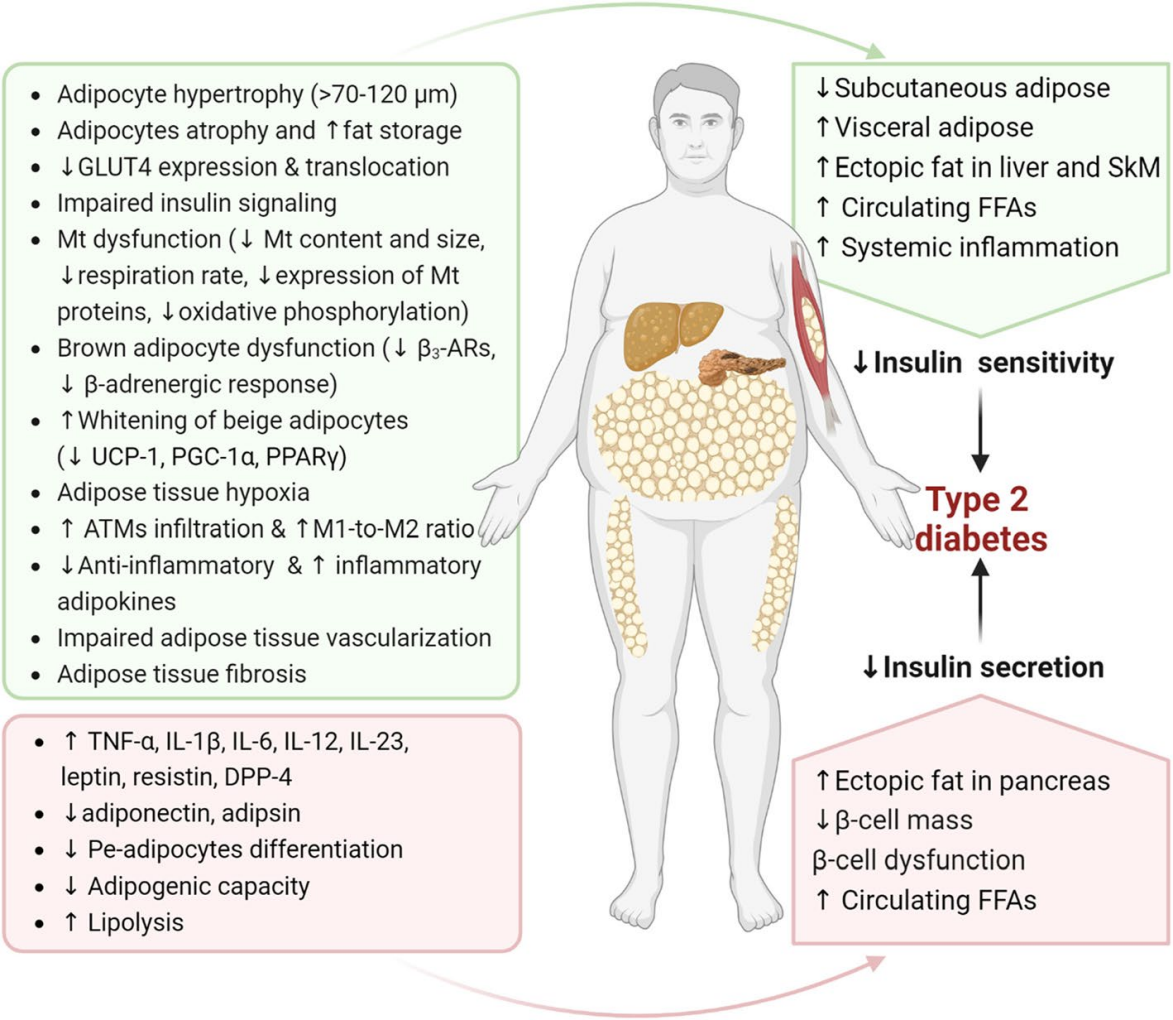
Major underlying mechanisms of dysfunctional adipose tissue leading to development of T2D. Green boxes address main mechanisms leading to systemic IR, and pink boxes underline mechanisms leading to β-cell dysfunction and decreased insulin secretion. Some mechanisms (e.g., those increase circulating FFAs) contribute in pathophysiology of T2D via both inducing IR and impaired insulin secretion. GLUT4, glucose transporter 4; FFA, free fatty acid; IL, interleukin; Mt, mitochondrion; PPARγ, peroxisome proliferator-activated receptor γ; T2D, type 2 diabetes; UCP1, uncoupling protein-1; PGC1α, peroxisome proliferator-activated receptor-γ coactivator 1-α; DPP4, dipeptidyl peptidase-4; ATM, adipose tissue macrophage; β_3_-AR, β_3_-adrenergic receptor; SkM, skeletal muscle; TNF-ɑ, tumor necrosis factor-ɑ

Several underlying mechanisms may link ATD to the development of T2D (Fig. 2). In brief, ATD manifested as fat maldistribution (i.e., increased visceral-to-subcutaneous fat ratio [31] and accumulated ectopic fat deposition, especially in the pancreas, skeletal muscle, and liver [32–34]) and adipose tissue remodeling (e.g., increased macrophage infiltration [35], inadequate vascularization [36, 37], increased collagen deposition and fibrosis [37–39], hypoxia [40, 41], altered adipokines and cytokines secretion [42], and mitochondrial dysfunction [43, 44]) leads to a systemic insulin resistance (IR) [45–50]. ATD leads to an excessive release of free fatty acids (FFAs), reactive oxygen species (ROS), and pro-inflammatory cytokines to the circulation, resulting in dysfunction of the main organs involved in the whole-body glucose metabolism (i.e., pancreas, liver, and skeletal muscle), leading to impaired insulin secretion (by impairing pancreatic β-cell dysfunction), systemic IR and establishing T2D [47–49]. Oxidative stress and specific markers of inflammation mediate, at least partly, the observed association between ATD and progression of Pre-DM since subjects with high-VAI values were found to have higher levels of C-reactive protein (CRP), interleukin-6 (IL‐6), tumor necrosis factor α (TNF‐α), serum amyloid‐A, homocysteine, and fibrinogen [51]. Elevated TNF-α levels lead to IR by inhibiting the expression of genes that are essential for insulin signaling and adipocyte differentiation [i.e., CCAAT-enhancer-binding protein-α, PPAR-g, glucose transporter type 4 (GLUT4), insulin receptor substrate-1 protein, adiponectin, and long-chain fatty acid acyl-CoA synthase] [52]. Severe ATD may accelerate the development of IR and T2D [53, 54], probably via overproduction of inflammatory cytokines and adipokines [55]. Excessive visceral adipose tissue accumulation may also increase the risk of IR and T2D via accelerated releasing FFAs [56, 57] and visfatin [58], upregulating pro-inflammatory cytokines, i.e., IL-6, TNF-α, and IL-1β, as well as disrupting the insulin-signaling pathways [59].

VAI represents physical and metabolic functions of adipose tissue, including altered production of adipocytokines, increased lipolysis, and plasma free fatty acids, which are not signified by simple anthropometric measures, including BMI, WC, and fat mass [7]. The ability of the VAI to express adipose tissue function goes beyond a hypothesis initially conceptualized based on some simple correlations with adipocytokines production [60, 61]. Of note, VAI outperforms common anthropometric and metabolic measures in predicting the 10‐year risk of T2D in the ATTICA study [51]. VAI values of more than 1.88 increased the risk of developing T2D by two-fold, independent of the well-known risk factors; a 22% elevated 10‐year risk of T2D was shown per one unit of VAI increase at baseline [51]. In a prospective study among the Chinese population, the highest VAI values (> 2.14 compared to < 1.14) increased the four‐year risk of T2D in men by about threefold (HR = 2.85, 95% CI = 1.81–4.48) [62]; VAI could also independently predict five- and 15‐year risk of T2D [63, 64]. Contrary to the BMI, WC, WHR, and WHtR, a direct association between VAI and risk of both pre-diabetes/diabetes was highlighted in a cross-sectional study comprising 2754 Chinese adults, independent of age, lifestyle variables, blood pressure, LDL-c, serum creatinine, serum uric acid levels and inflammatory markers [65]. It has been highlighted that the VAI could considered as an indicator of adipose distribution and function that indirectly expresses cardiometabolic risk and be a useful index for early detection of cardiometabolic risk before its development [11].

The main strength of this prospective study lies in the reliable follow-up in a well-characterized population-based representative sample of Pre-DM subjects in which the outcomes have been documented with standardized measures both at baseline and follow-up examinations and systematically recording the variables required for VAI calculating and completeness of ascertainment and accuracy of ATD classification. However, the findings’ interpretation should be undertaken within the context of the potential limitation of our study. Due to potential changes in dietary intakes and other T2D risk factors during the study follow-up, some degree of misclassification might have occurred, which could lead to biased estimated odds ratios towards the null, as inherent in any prospective study. Furthermore, the association between ATD and Pre-DM progression to T2D may be affected by genetic polymorphisms related to T2D. In our study, we could not perform Mendelian randomization to consider potential genetic variants in relation to ATD and T2D and provide an unbiased ‘causal’ estimate of the effect(s) of severe ATD on the risk of Pre-DM progression. We also could not consider other potential confounding factors, e.g., alcohol consumption, sociodemographic status, and food security, in our statistical models.

In conclusion, our findings suggested that ATD, defined based on the age-stratified cutoff values of VAI as a reliable indicator of visceral fat metabolism, has a predictive performance for future glycemic status of Pre-DM subjects. Severe ATD may increase the risk of Pre-DM progression to T2D and reduce the chance of returning to normoglycemia. ATD may be a simple and practical index for detecting subjects at a higher risk of Pre-DM progression to T2D, allowing for timely intervention strategies.

## Data Availability

The datasets generated and/or analyzed during the current study are not publicly available, but will be available by the corresponding author (z.bahadoran@endocrine.ac.ir) on reasonable request after confirmation of the director of RIES (azizi@endocrine.ac.ir).

## Abbreviations

Pre-DM: Prediabetes
T2D: Type2 Diabetes
CI: Confidence Interval
OR: Odds Ratio
TLGS: Tehran Lipid and Glucose Study
WC: Waist Circumference
BMI: Body Mass Index
SBP: Systolic Blood Pressure
DBP: Diastolic Blood Pressure
FSG: Fasting Serum Glucose
2h-SG: 2-Hour Serum Glucose
IFG: Impaired Fasting Glucose
IGT: Impaired Glucose Tolerance
NFG: Normal Fasting Glucose
NGT: Normal Glucose Tolerance
TG: Triglyceride
HDL-c: High-Density Lipoprotein cholesterol
OGTT: Oral Glucose Tolerance Test
MAQ: Modifiable Activity Questionnaire
FFQ: Food Frequency Questionnaire
RR: Relative risk
HR: Hazard ratio
VAI: Visceral adiposity index
ATD: Adipose tissue dysfunction
NCD: Non-communicable disease
FHD: Family history of T2D
IQR: Inter-quartile range
CRP: C-reactive protein
IL‐6: Interlukine-6
TNF‐α: Tumor necrosis factor α
